# Supplementary material for: Clinical outcomes in patients with muscle disorders and acute ischemic stroke
Source: PLoS One. 2025 Aug 12;20(8):e0329631. doi: 10.1371/journal.pone.0329631 (PMC12342303; doi:10.1371/journal.pone.0329631)
Supplement: S1 Table — (DOCX) [file pone.0329631.s001.docx]

**Supplementary Table 1**. ICD-10 codes and NIS data used for definitions of variables.

| **Identifier** | **Category** | **Variable** | **Code** |
| --- | --- | --- | --- |
| ICD-10-CM | Inclusion  criteria | Ischemic stroke | I63x[15, 16] |
| NIS Reported[17] | Inclusion  criteria | Age | - |
| NIS Reported[17] | Inclusion criteria | Non-elective admission status | - |
| NIS Reported[17] | Outcome | In-hospital death | - |
| ICD-10-CM | Outcome/  covariate | NIH Stroke Scale | R29.7x[18] |
| ICD-10-PCS | Outcome/  covariate | Endovascular thrombectomy for acute ischemic stroke | 03CG3Z7, 03CG3ZZ, 03CG4Z6, 03CG4ZZ, 03CH3Z6, 03CH3Z7, 03CH3ZZ, 03CH4Z6, 03CH4ZZ, 03CJ3Z6, 03CJ3Z7, 03CJ3ZZ, 03CJ4Z6, 03CJ4ZZ, 03CK3Z6, 03CK3Z7, 03CK3ZZ, 03CK4Z6, 03CK4ZZ, 03CL3Z6, 03CL3Z7, 03CL3ZZ, 03CL4Z6, 03CL4ZZ, 03CP3Z6, 03CP3Z7, 03CP3ZZ, 03CP4Z6, 03CP4ZZ, 03CQ3Z6, 03CQ3Z7, 03CQ3ZZ, 03CQ4Z6, 03CQ4ZZ[16] |
| ICD-10-PCS/  DRG | Outcome/  covariate | Intravenous thrombolysis | 3E03317, DRG 61-63[16, 19] |
| ICD-10-CM | Exposure | Muscle disorder | ICD: G71-72 |
| Elixhauser comorbidity score[20-22] | Covariate | Hypertension | - |
| Elixhauser comorbidity score[20-22] | Covariate | Diabetes | - |
| Charlson comorbidity score[20-22] | Covariate | Congestive heart failure | - |
| Elixhauser comorbidity score[20-22] | Covariate | Obesity | - |
| ICD-10-CM | Covariate | Atrial Fibrillation | I48x[23, 24] |
| NIS Reported[17] | Covariates | Hospital Census region, patient urban-rural residence, hospital teaching status and bed size | - |
