# Supplementary material for: Clinical outcomes in patients with muscle disorders and acute ischemic stroke
Source: PLoS One. 2025 Aug 12;20(8):e0329631. doi: 10.1371/journal.pone.0329631 (PMC12342303; doi:10.1371/journal.pone.0329631)
Supplement: Attachment — Submitted filename: Response to Reviewers - PONE-D-24-54767.docx [file pone.0329631.s003.docx]

March 5, 2025

We would like to thank the Editorial Board and Reviewers for the opportunity to submit a revised version of “Clinical outcomes in patients with muscle disorders and acute ischemic stroke” to *PLOS ONE*. We have responded to the comments below.

**Reviewer #1:** This is an interesting study. There is novelty in this research. I highlight some methodological flaws in design. I give some comments and suggestions:

1. Please be detail in the method. Is it retrospective, cross sectional, or cohort ? Cross sectional is smash-spot study. In table, the authors use cohort. Please be consistent.

*Our study is retrospective, and we have revised the manuscript in all relevant places to state this. We keep use of the word “cohort” to describe our study population, such as in places like the table.*

2. The method should highlight the definition of muscle disease, the type of muscle disease, the specific treatment.

*We already included which muscle disorders were our primary exposures in the methods section and listed their corresponding ICD-10 codes (G71-72). We did not detail specific treatments for each condition in the methods section as we felt this would distract from the focus of our study and analysis. However, we have included a short paragraph describing the treatment of muscle disorders in the introduction as background information.*

1. The discussion should begin with the findings of your study. The discussion should elaborate more the findings of the study.

*Thank you for the comment. We have reiterated findings more explicitly.*

2. The discussion should highlight more the mechanism and biological plausibility of this findings.

We have added in that the mechanism of these worse outcomes can be potentially secondary to patient baseline weakness and poor functional reserve, leading to less likelihood of being discharged home and having increased risk of mortality.

3. The conclusion should answer the clinical question. The conclusion should be concise and clear.

We have added a conclusion section with a succinct and clear line about the findings and answering our clinical question about outcomes of these patients.
